# An Ancient Molecular Arms Race: *Chlamydia* vs. Membrane Attack Complex/Perforin (MACPF) Domain Proteins

**DOI:** 10.3389/fimmu.2020.01490

**Published:** 2020-07-14

**Authors:** Gabrielle Keb, Kenneth A. Fields

**Affiliations:** Department of Microbiology, Immunology & Molecular Genetics, University of Kentucky College of Medicine, Lexington, KY, United States

**Keywords:** immunity, evolution, pathogenesis, pore-forming, obligate intracellular

## Abstract

Dynamic interactions that govern the balance between host and pathogen determine the outcome of infection and are shaped by evolutionary pressures. Eukaryotic hosts have evolved elaborate and formidable defense mechanisms that provide the basis for innate and adaptive immunity. Proteins containing a membrane attack complex/Perforin (MACPF) domain represent an important class of immune effectors. These pore-forming proteins induce cell killing by targeting microbial or host membranes. Intracellular bacteria can be shielded from MACPF-mediated killing, and *Chlamydia* spp. represent a successful paradigm of obligate intracellular parasitism. Ancestors of present-day *Chlamydia* likely originated at evolutionary times that correlated with or preceded many host defense pathways. We discuss the current knowledge regarding how chlamydiae interact with the MACPF proteins Complement C9, Perforin-1, and Perforin-2. Current evidence indicates a degree of resistance by *Chlamydia* to MACPF effector mechanisms. In fact, chlamydiae have acquired and adapted their own MACPF-domain protein to facilitate infection.

## Introduction

Obligate intracellular bacteria depend on survival within eukaryotic host cells. The family *Chlamydiaceae* contains at least nine designated species of obligate intracellular pathogens exhibiting a diverse host range in higher eukaryotes. *C. trachomatis* and *C. pneumoniae* represent species commonly impacting human health. *C. pneumoniae* infects the upper respiratory tract and is associated with 10–20% of adult community-acquired pneumonia (1). *C. trachomatis* urogenital infection (serovars D-K) continues to be the most common sexually transmitted bacterial infection in the US (2) and ocular infection (serovars A-C) is the leading cause of infectious blindness in developing countries (3). Interestingly, anecdotal evidence suggests that chlamydial ocular infections have affected humans for millennia (4). Regardless of species, all *Chlamydia* share a biphasic developmental cycle that alternates between infectious elementary bodies (EBs) and non-infectious reticulate bodies (RBs). EBs have minimal metabolic activity and are often referred to as “spore-like” due to a durable cell wall that is resistant to mechanical and osmotic pressures [reviewed in (5)]. EB envelopes are comprised of an “atypical” Gram-negative lipid bilayer that is stabilized through disulfide bonds among cysteine-rich outer membrane proteins [reviewed in (6)]. During invasion, EBs traverse the host-cell plasma membrane and establish an intracellular niche within a membrane bound vesicle termed the inclusion. Once the inclusion is established, EBs differentiate into non-infectious RBs which are capable of robust protein synthesis and replication. This stage of the developmental cycle occurs entirely within the protection of the infected host cell. The cycle is completed by asynchronous differentiation of RBs back into EBs capable of infecting neighboring cells after release. Escape of EBs from the host cell is accomplished by either lysis of the host cell or extrusion of intact inclusions (7).

The *Chlamydiales* order also contains *Chlamydia*-related bacteria often referred to as environmental *Chlamydia* due to their obligate intracellular parasitism of amoeba (8). The *Chlamydiaceae* family diverged from *Chlamydia*-related bacteria an estimated 700 million years ago at a time when all eukaryotes were single cell [reviewed in (9)]. Due to a dependence on intracellular survival and long evolution with eukaryotic hosts, it is not surprising that *Chlamydia* may have adapted multiple immune evasion strategies. During *C. trachomatis* genital infection, both innate and adaptive immune responses are clearly elicited with innate immunity functioning to limit ascension of infection and a Th1-, IFNγ-dependent adaptive response being required for control and resolution [reviewed in (10)]. Reinfection is common, and the majority of infections are asymptomatic, particularly in women (11). Progress in developing an efficacious vaccine has been challenged by poor protective immunity and increased pathology (12). Detrimental patient outcomes, such as tubal factor infertility and pelvic inflammatory disease, are associated with severe immunopathology which is initiated by the infected epithelium (13). Taken together, it is evident that *Chlamydia* maintain a finely tuned relationship with their host to interfere with productive immune recognition and clearance.

Formation of targeted membrane spanning pores using membrane attack complex/Perforin (MACPF)-domain proteins represents one mechanism used by both innate and adaptive arms of immunity. The MACPF-containing host proteins Perforin-2 and Complement C9 represent innate immune effectors whereas Perforin-1 functions during adaptive immunity (14). Both C9 and Perforin-2 are evolutionarily ancient whereas Perforin-1 likely arose by gene duplication of Perforin-2 during evolution of adaptive immunity in multi-cellular organisms (15, 16). All three function by killing microbes (C9 and Perforin-2) or host cells (Perforin-1) via polymerization and pore formation in target membranes (17). Interestingly, chlamydial genomes also contain a gene encoding a MACPF domain protein. It is hypothesized that this domain was acquired through horizontal gene transfer with a mammalian host (18, 19). A recent metagenomic study found only a small number of protein families that were taxonomically restricted within *Chlamydiaceae* (20). MACPF-containing proteins were among factors related to specific host interactions, providing further evidence that *Chlamydia* likely acquired this domain through co-evolution with a mammalian host.

Given the apparent long co-evolution of the host-pathogen interaction exemplified by *Chlamydia*, this review will summarize current evidence of chlamydial resistance and susceptibility to MACPF domain-mediated attack strategies while highlighting immune evasion mechanisms adapted through co-evolution. The discussion will focus on the more thoroughly characterized human pathogen *C. trachomatis* and corresponding immune modeling in mice using *C. muridarum*. We will also discuss the implications of the endogenous chlamydial MACPF domain protein in infection biology.

## The C9 MACPF Domain

Complement is a central defense mechanism of the innate immune system that evolved to inactivate extracellularly localized pathogens (16). The complement system consists of more than 30 soluble serum proteins culminating in formation of the membrane attack complex (MAC) for complement-mediated cell lysis. The pore forming complex targets outer membranes of Gram-negative bacteria, enveloped viruses, and parasites. Activation of the complement cascade can occur via lectin, classical, or alternative pathways. Each pathway differs in the early mechanism used to recognize pathogens, but all converge through the covalent attachment of C3b to the target cell which then recruit downstream factors leading to MAC formation. Complement activation clearly occurs during chlamydial infection with the antibody-independent alternative pathway playing a major role (21). Multiple studies using a tissue-culture infection model have demonstrated that *Chlamydia* inclusion formation is significantly inhibited when EBs are pre-incubated with normal human sera (21–25) indicating complement factors may be important for controlling infection.

After typical C3b deposition on a bacterial surface, subsequently recruitmented components C5b-C8 then recruit soluble C9 monomers and facilitate C9 polymerization and the assembly of an 88-strand β-barrel membrane spanning pore (26). CryoEM studies have revealed that active pores contain 6 poly peptide chains, C5b, C6, C7, C8a, C8β, and C8γ, with 18 C9 monomers (27, 28). In experiments using C3 deficient mice, *C. muridarum* infectivity was not impacted during genital infections (29), however, *C. psittaci* pneumonia was significantly exacerbated when chlamydiae were introduced via a respiratory route (30). These data raise the possibility that C5b-C8 recruitment of C9 and pore formation may lead to fatal disruption of the chlamydial envelope; yet, formation of the MAC appears to be dispensable as a primary control mechanism for *Chlamydia* infections. Depletion of factors C5 and C8 from serum had no effect on *in vitro* anti-chlamydial activity (24). Additionally, *C. muridarum* shedding and ascension into the upper genital tract was not impacted in C5-deficient mice (29). Together these data indicate that late complement factors do not play a significant role in directly inactivating *Chlamydia*. The anaphylatoxin activities of C5a and C3a have been proposed as mediators of complement-dependent effects on infectivity and pathogenesis in mice (29–31). The antibody-independent inhibitory activity of complement in human serum could be mediated by opsonization and inactivation of chlamydial surface proteins required for cellular attachment and invasion (21), or deposition of other components such as properdin leading to targeting of *Chlamydia* to the lysosomal pathway for degradation (32). It remains undefined, however, how findings in cell culture are related to those observed *in vivo*. Clearly, further investigation of this interesting area is warranted.

Given that the complement system primarily targets extracellular invaders, the obligate intracellular lifestyle of all *Chlamydia* spp. represents the most obvious defense mechanism. Beyond that, the biphasic developmental cycle represents an additional layer of protection. The extracellular, infectious EB possess a rigid and highly disulfide crosslinked outer envelope. Assembly of the MAC requires a fluid membrane capable of allowing lateral diffusion of MAC components and dramatic structural changes associated with pore formation (33). We therefore speculate that the EB envelope would be impervious to the MAC. A highly conserved chlamydial protease, CPAF, cleaves C3, and factor B *in vitro* and may inhibit activation of the alternate complement pathway (25). Host cell escape through extrusion, one of two chlamydial exit strategies, may be another defense mechanism. During extrusion, the inclusion pinches off from the host cell in an exocytosis-like mechanism. Both the host cell and inclusion remain intact, and the now double-membrane-bound inclusion is released into the extracellular space where it is stable up to 4 h *in vitro* (7, 34–36). We predict that it is unlikely that complement factors could gain access through the multiple layers of membrane comprising this barrier. *Chlamydia*-mediated recruitment of CD59 to the inclusion membrane (37) would represent an additional layer of defense. CD59 regulates formation of the MAC to prevent uncontrolled complement-mediated cell lysis (38) and would prevent any lytic pore formation should barrier integrity be compromised.

## Perforin

The cytotoxic functions of natural killer cells (NKs) and cytotoxic T lymphocytes (CTLs) represent an adaptive defense mechanism against viral and intracellular pathogens. NKs and CTLs release cytoplasmic granules containing Perforin and proteolytic granzymes onto the surface of infected cells. In the presence of Ca^2+^, Perforin binds to the target membrane and forms a transmembrane β-barrel pore. The N-terminal domain of Perforin contains a MACPF domain that allows insertion into lipid bilayers (39). Once assembled, the Perforin pore functions to deliver the proteolytic granzymes to the cytosol of the targeted cell. Two models of Perforin mediated granzyme delivery exist. The original model proposes that the Perforin pore provides direct delivery of granzymes to the cytosol, and a second model proposes that both Perforin and granzymes are endocytosed into the cell with subsequent delivery of granzymes by Perforin (40, 41). This model is supported by data demonstrating Perforin alters membrane curvature and stimulates the formation of endocytic vesicles (42).

During respiratory infection with *C. muridarum*, NKs infiltrate the lungs and become activated (43). Multiple studies have shown that NKs contribute modestly to clearance of chlamydial infection, however, this effect may be driven by IFN-γ expression and independent of Perforin targeting (43–45). *Chlamydia* infected cells are highly resistant to induction of apoptosis which is predicted to be due to chlamydial proteins that interrupt events such as cytochrome C release from mitochondria (46). NK cells extracted from *C. trachomatis* infected patients have also be shown to have decreased lytic capability (47). In two studies, Perforin knockout mice were not compromised in their ability to clear *C. muridarum* genital infection (48, 49), indicating Perforin-mediated cytotoxicity is not required for clearance of primary chlamydial infection. A third study, using lower infectious doses, did note a delayed clearance of infection in Perforin -/- mice (50); however, the authors concluded from their additional data that the phenotype occurred independently of direct interaction of cytotoxic cells with infected epithelia. Finally, the IFN-γ dependent/Perforin independent clearance of *Chlamydia* is supported by the finding that NK cells have a differential effect during infection where IFN-γ production is increased, yet cytolytic function is decreased (43).

Although some studies indicated *Chlamydia*-mediated interference with inducible expression of class I MHC on infected cells (51), primary chlamydial defense against Perforin may be more passive. *Ex vivo* studies indicated that *Chlamydia*-infected epithelial cells can be lysed by cytotoxic cells (52–54). Whether host cell lysis would directly contribute to control of chlamydial infection, however, is unclear since the disrupted cells would merely release any infectious EBs that had formed. Indeed, *in vivo* work noted above is consistent with Perforin-independent control mechanisms. In addition, the female genital tract represents one location where tolerance to foreign antigens must be greater for sustainment of the natural microbial flora. In both the gastrointestinal and female genital tracts, CD8+ T-cells have decreased expression of Perforin, thus comparatively limited cytotoxic activity (8, 55). In endocervical samples from both non-infected and *C. trachomatis* infected patients, effector memory T cell subsets showed decreased Perforin expression as compared to paired blood controls (55). Therefore, infection of the genital tract likely provides an advantageous niche for chlamydial infection.

## Perforin-2

In contrast to the relative lack of susceptibility of *Chlamydia* to Perforin-1 and Complement C9, there does appear to be a role for the most recently described MACPF protein, Perforin-2. Perforin-2, encoded by the intronless *MPEG1*, represents perhaps the most evolutionary ancient and conserved member of the MACPF family of proteins (16, 56, 57). Originally shown to have anti-bacterial properties in sponges (58) and zebrafish (59), it is now established that Perforin-2 is capable of killing a range of cell-associated bacteria including Gram positive, Gram negative, and acid fast bacteria (60). A model has emerged where Perforin-2 is trafficked to bacteria-containing vacuoles and disrupts the integrity of bacterial envelopes by polymerizing into multi-subunit pores (14, 57). To date, the susceptibility of *Chlamydia* spp. to Perforin-2 represents the sole indication for how an obligate intracellular bacterium might respond to this novel innate immune mechanism (61).

Professional phagocytes, including macrophages and neutrophils, represent a functionally important arm of innate immunity, and Perforin-2 expression is constitutive in these cells (60). Cumulative data using murine-specific *C. muridarum* in a well-established mouse model of genital tract infection indicate robust recruitment of professional phagocytes to infected tissues. Although innate immunity is not required for resolution of infection, it has been proposed to function in opposing ascension of chlamydial infection into the upper genital tract (10). Although some degree of chlamydial growth can be detected in macrophage cell lines, *Chlamydia* spp. do not productively infect primary cells (62). RNAi knock-down of *MPEG1* message was used to provide direct evidence for Perforin-2-mediated eradication of *Chlamydia* in infected macrophages (61). Transmission electron microscopy revealed that mock-treated murine BV2 macrophages contained vacuoles harboring debris and few intact chlamydiae, whereas Perforin-2-deficient cells contained intact inclusions that yielded 10^3^ more progeny 24 h post infection. Knock-down of Perforin-2 resulted in levels of progeny *C. trachomatis* L2 EB production equivalent to similarly infected HeLa cells. Comparable results were seen for *C. trachomatis* serovars B and D indicating that Perforin-2 is capable of inhibiting a range of *C. trachomatis* serovars. In addition, *C. muridarum* was also susceptible to Perforin-2 activity. The BV2 line was initially tested due to the comparatively high level of constitutive Perforin-2 expression, yet similar results were obtained using the murine RAW 264.7 cell line (61). To date, the potential role of Perforin-2-dependent inhibition of *Chlamydia* has not been tested in human cell lines such as THP-1 or HL-60; however, Perforin-2 has been shown in these cells to limit growth of other bacteria such as *Salmonella, S. aureus*, and *Mycobacterium* (60) raising the probability that the observed anti-*Chlamydia* potential of Perforin-2 also occurs in humans cells.

These data are consistent with macrophage-produced Perforin-2 having a significant role in controlling chlamydial infection. How Perforin-2 inactivates intra-inclusion chlamydiae remains an open question. *Chlamydia* are rapidly targeted to lysosomal compartments in macrophages (61), yet develop normally in Perforin-2 deficient macrophages. Hence, Perforin-2 plays an active role in clearance at the cellular level. Based on a proposed working model for susceptibility of intracellular bacteria (14), deployment of Perforin-2 to the luminal face of subsequent *Chlamydia*-containing vacuolar membranes would culminate in insertion and polymerization of the Perforin-2 pore in chlamydial membranes. This would be predicted to disrupt the integrity of the RB envelope and lead to lysis. This model is supported by electron micrographs of Perforin-2 sufficient and deficient cells were clearly lysed chlamydial material is detected within an apparently intact vacuole (61). The *Chlamydia* highly cross-linked envelope of the EB developmental form (6) is likely resistant to Perforin-2 insertion. However, Perforin-2-mediated killing of *Mycobacteria* spp., which possess a highly impermeable, mycolic acid-containing envelope, suggest that the atypical RB envelope may be susceptible to Perforin-2 attack. Perforation of bacterial envelopes has also been shown to promote access of antimicrobial substances like reactive oxygen species (63). This mechanism may contribute to observed *Chlamydia* clearance since RAW 264.7 macrophages generate ROS and iNOS in response to infection, and pharmacologic inhibitors or scavengers of reactive species benefit chlamydial survival (64). Finally, macrophage-mediated killing of *C. trachomatis* has also been linked to autophagy (65–67). It is possible that Perforin-2 and autophagy mechanisms are linked, yet Perforin-2 has been proposed to function upstream of autophagy based on the greater impact on chlamydial survival (61).

Interestingly, chlamydial exit from infected epithelial cells via extrusion may subvert Perforin-2-mediated killing. *Chlamydia*-containing extrusionsare phagocytosed by bone marrow-derived macrophages and retain infectivity compared to non-encapsulated *Chlamydia* (36). In this scenario, the extra lipid bilayer could shield *Chlamydia* from detection and Perforin-2-mediated killing since machinery necessary for targeting Perforin-2 to membranes would be absent from the extrusion membrane (68) Finally, *C. pneumoniae* appears to be capable of replication in a subset of primary phagocytes (62), raising the possibility of additional, species-specific protective mechanisms. This would be consistent with observations that other pathogens such as *Salmonella* and enteropathogenic *E. coli* have evolved Perforin-2-specific mitigation mechanisms (68).

Columnar epithelial cells lining target mucosa represent the productive replication niche for all *Chlamydia* spp. Therefore, *Chlamydia* would be predicted to possess effective Perforin-2 protective mechanisms in this cell type. *MPEG1* is inducible in non-myeloid cells, and Perforin-2-specific signal is below detection in multiple epithelial lines (60). Indeed, Perforin-2 was absent in both mock-treated and *Chlamydia*-infected cells in a HeLa-cell infection model (61). In contrast, treatment of cells with heat-killed *Chlamydia* resulted in significant up-regulation of Perforin-2, indicating that stealthy subversion of signals leading to *MPEG1* expression as one protective mechanism. *MPEG1* is inducible with IFNγ and type I interferons (60), both of which play important roles in limiting chlamydial infection *in vivo* (10). Indeed, *MPEG1* upregulation was evident in early microarray analyses of IFNγ-treated oviduct epithelial cells (69). Interestingly, cells containing established inclusions prevent IFNγ-mediated induction of Perforin-2 in epithelial cells. This effect required viable *Chlamydia*. The role of IFNβ-mediated induction of *MPEG1* during chlamydial infection has not been tested, yet a recent report indicated a requirement of Perforin-2 in transducing activation signals through the cognate receptor, IFNAR (70). Hence, Perforin-2 mediated killing is counter-indicated in infected epithelial cells. Importantly, *ex vivo* treatment of murine genital tract-derived epithelial cells with *Chlamydia*-conditioned media resulted in upregulation of *MPEG1* (50). Proinflammatory signals are certainly capable of acting on uninfected, bystander cells which could then be more resistant to chlamydial infection in a Perforin-2-dependent manner. In support of this notion, ectopic overexpression of RFP-tagged Perforin-2 in HeLa cells resulted in efficient killing of *Chlamydia* (61). It is important to note that polymerization of Perforin-2 for crystallographic studies required an acidic environment and is postulated to reflect delivery of Perforin-2 to acidified phagolysosomes (71, 72). The chlamydial inclusion, however, does not acidify and remains segregated from the lysosomal pathway (73), raising the possibility of alternative polymerization mechanisms. It is also possible that non-physiologic levels due to ectopic expression favor polymerization *in vivo*. Regardless, it seems apparent that *Chlamydia* avoid Perforin-2 killing in epithelial cells by avoiding and suppressing *MPEG1* expression. Although Perforin-2 proteins levels were not shown, inhibition of *C. muridarum* in IFNγ-primed murine embryonic fibroblasts was unchanged after Perforin-2 knockdown using siRNAs (74). These data may, once again, elude to alternative, species-specific susceptiblity of *Chlamydia* to Perforin-2.

**Figure 1:**
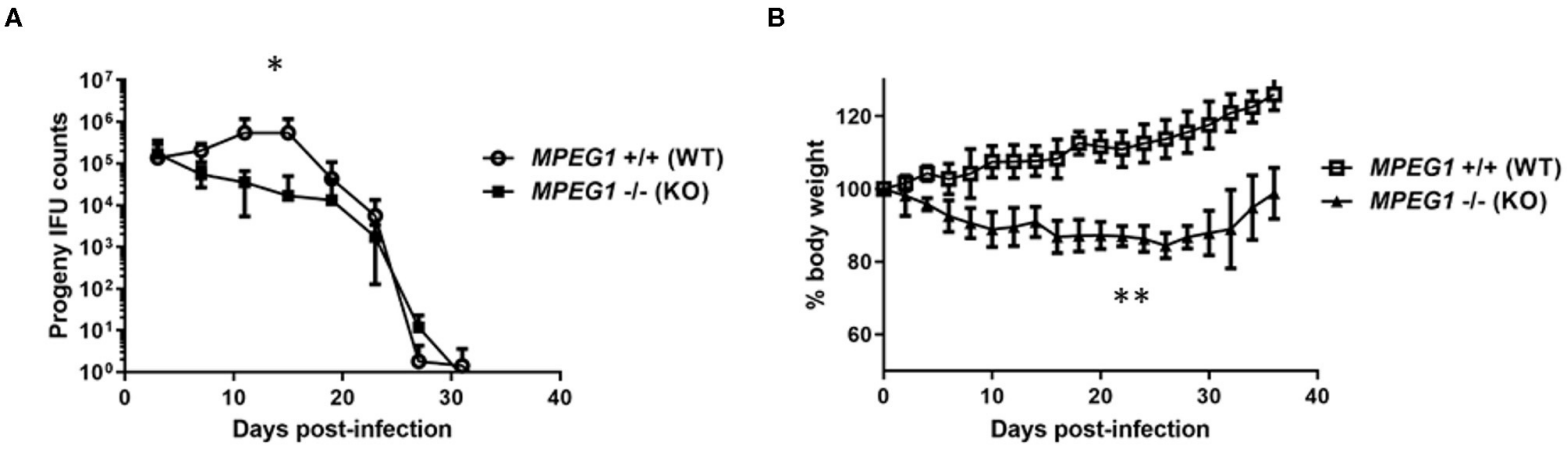
*C. muridarum* infection of Perforin-2 KO mice. Groups of 5, 6–7 week-old female wild type (*MPEG1*+/+) or KO (*MPEG1* -/-) C57BL/6 × 129 × 1/SJV mice (60) were infected intravaginally with 5 × 10^4^ *C. muridarum* 5 days after synchronization with medroxyprogesterone. Shed IFUs **(A)** and body weights **(B)** were measured over time (days). **(A)** Shed IFUs were enumerated every 4 days according to standard protocols and averages within groups are shown with standard deviations. Curves were different (*p < 0.01) by two-way ANOVA. **(B)** Average body weights (+/– standard deviation) within groups are shown. KO weights were significantly different (**p < 0.0001) from WT via two-way ANOVA. Data represent one of two replicate experiments (*n* = 2).

Mice deficient in *MPEG1* have subsequently been employed to test the anti-bacterial role of Perforin-2 in an animal model. Cells derived from *MPEG1* -/- mice are deficient in *ex vivo* killing of a range of bacteria including *L. monocytogenes* (75), *Mycobacteria* spp., *S. aureus, S. enterica* Typhimurium, and enteropathogenic *E. coli* (60, 68). *MPEG1* -/- mice are more susceptible to systemic listeriosis (75) and succumb to sublethal doses of *S. aureus* and *Salmonella* in cutaneous and orogastric infection models, respectively (60). Collectively, these data indicate the fundamental importance of Perforin-2 in controlling a diverse array of bacterial infections. It remained unclear how the impact of Perforin-2 would manifest during a mammalian model of chlamydial infection. To that end, the well-established intravaginal infection model was employed were *Chlamydia* are deposited at the cervical vault and bacterial shedding is enumerated by swabbing over time. We infected groups of 5 wild type or *MPEG1* -/- mice with 5 × 10^4^ infectious particles of *C. muridarum* and monitored chlamydial shedding and mouse body weight over time (Figure 1). Time to resolution was not extended in the absence of Perforin-2. However, MPEG -/- mice shed statistically fewer IFUs overall with a ca. log_10_ decrease observed on days 11 and 15. Interestingly, the *MPEG1* -/- mice did appear ill with ruffled fur (Fields, unpublished) and decreased body weight (Figure 1B). These symptoms persisted until times corresponding to resolution. We speculate that these data could indicate systemic dissemination of chlamydiae beyond the genital tract. Infiltration of macrophages and neutrophils to the sites of active infection functions to contain chlamydiae (10). Less efficient killing by professional phagocytes could allow rapid ascension and seeding of peripheral sites. The role of these myeloid cells could be easily tested using bone-marrow chimeric mice expressing or lacking *MPEG1*. A functional adaptive response is likely intact given that infections in *MPEG1* -/- mice were resolved comparable to wild type. As is the case with the molecular mechanisms of Perforin-2 function in cell culture, many provocative questions remain to be resolved.

## The Chlamydial MACPF

**Figure 2:**
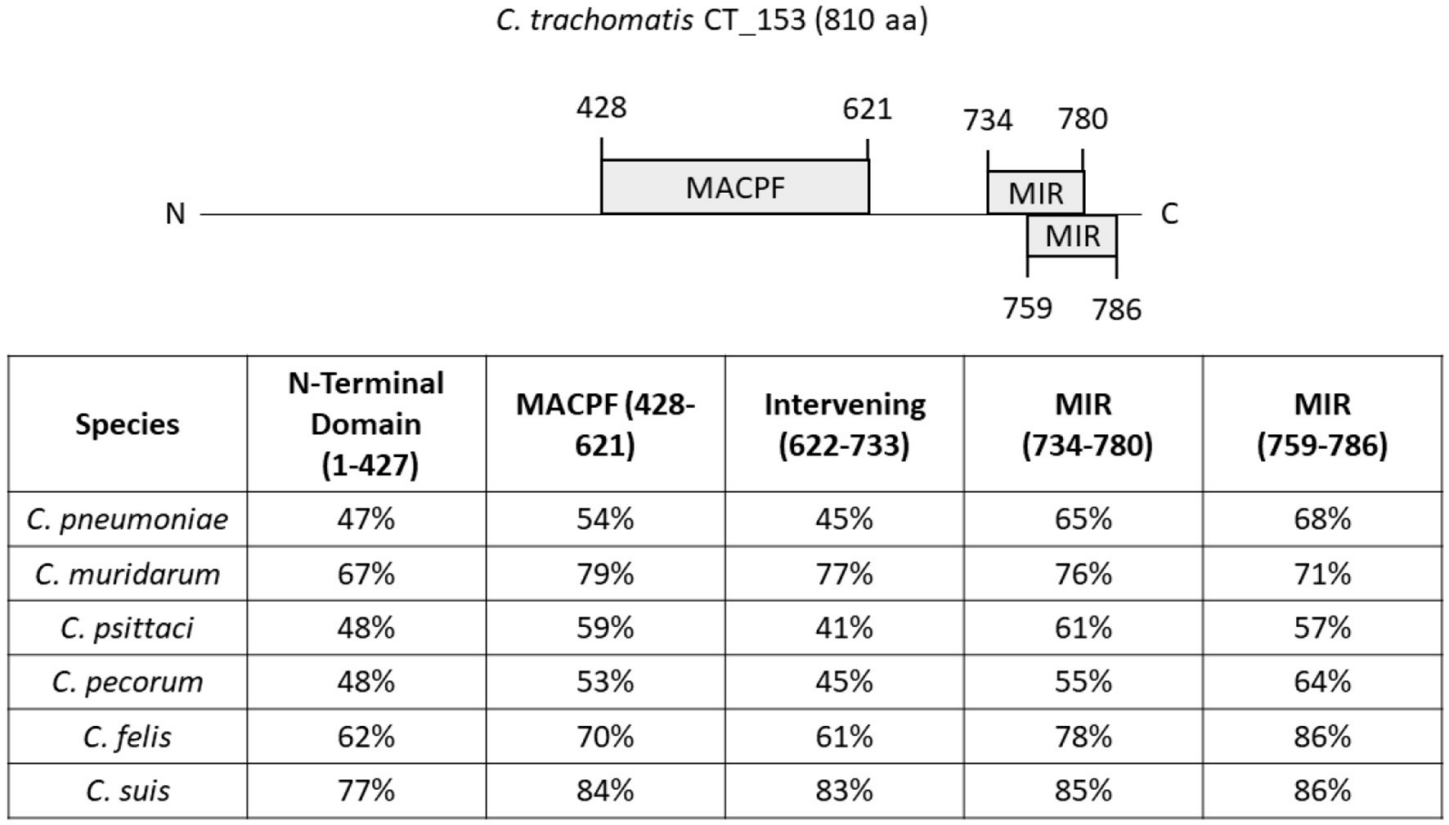
Similarity of *C. trachomatis* CT153 domains to orthologs in other *Chlamydia* spp. The 810 residue CT153 of *C. trachomatis* D/UW-3 is shown schematically with predicted functional domains highlighted. These include the MACPF and Mannosyltransferase, Inositol 1,4,5-trisphosphate receptor and Ryanodine receptor (MIR) domains. Residues corresponding to the N-Terminal domain (aa 1-427), MACPF (aa 428-621), Intervening domain (aa 622-733), or MIR (aa 734-780 and aa 759-786) were used as query sequences in NCBI BLAST searches. Searches were performed using *C. pneumoniae* TW-183, *C. psittaci* 6 BC, *C. muridarum* Nigg, *C. suis* S45, *C. felis* DSM:26967, and C. *pecorum* Bo/E58 data bases. Calculated percent identity in respective species is shown for each domain.

Whole-genome sequencing of *Chlamydia* spp. originally identified a gene encoding a MACPF domain protein (18). In the reference strain *C. trachomatis* serovar D, this protein is designated CT153 (76). Gene orthologs were conserved in all *C. trachomatis* genomes sampled and located within the highly variable plasticity zone, immediately upstream pzPLD genes encoding putative lipid-modifying proteins (77, 78). Although similarity varies, orthologs are also present in other *Chlamydia* spp (Figure 2). Consistent with acquisition through horizontal gene transfer during co-evolution, the MACPF gene sequence of *C. pneumoniae* can differentiate bacteria isolated from indigenous and non-indigenous human sources from varying geographical regions (79). C-terminal amino acid residues 427–621 share homology with the MACPF domain found in C9 and Perforin. CT153 appears to undergo some proteolytic processing (78). The full length p91 was observed as the dominant polypeptide in EBs and is rapidly cleaved (15 min) into p57 and p41 fragments independent of *de novo* chlamydial protein synthesis. Processing may occur through host-mediated proteolytic cleavage or auto-proteolysis. This suggests distinct functions for full-length and processed peptides, yet it should be noted that the possibility of post-lysis degradation has not been ruled out (80). Currently, there is limited understanding regarding the function and cytolytic activity of this protein. It was originally postulated that the MACPF domain protein may be essential for *Chlamydia* since a saturating screen for chemically-induced mutations in *C. muridarum* failed to reveal nonsense mutations in *tc0431* (81). However, inactivating transposon insertions were subsequently observed in both *C. muridarum* and *C. trachomatis* (82, 83). None of the mutations abolished intracellular survival, indicating that this protein is not essential for cultivation in tissue culture. These data are in line with the apparent lack of a MACPF-encoding gene in *C. abortus* which can infect the same cells (84). CT153 is expressed during mid-cycle (85) and has been shown to localize with both RBs near the inclusion membrane and within the inclusion membrane (78). These data suggest that CT153 may permeabilize the inclusion membrane. Complete lysis of the inclusion membrane is likely retarded by structural integrity conferred by chlamydial inclusion membrane proteins (86). For example, loss of specific Incs results in premature lysis of the parasitophorous vacuole (87, 88). The embedded pore could instead facilitate diffusion of small molecules into or out of the inclusion or be involved in fusion events during exocytic exit from cells. Clearly, this is a rich area needing further investigation.

## Concluding Remarks

*Chlamydia* are ancient bacteria that have evolved with mammalian hosts for of millions of years. To sustain a privileged niche and intracellular survival, *Chlamydia* have adapted resistance mechanisms to major host defenses that include formation of the MAC and Perforin mediated cytotoxicity. *Chlamydia* are not completely resistant to MACPF attack strategies since these pathogens are susceptible to Perforin-2 activities of professional phagocytes. Finally, acquisition of the MACPF domain from hosts may have given the bacteria an edge for survival, however it is unclear whether this domain is responsible for resistance to host immunity or has other functions.

## Author Contributions

Article preparation and writing was performed by GK and KF. KF performed work associated with Figure 1. All authors contributed to the article and approved the submitted version.

## Conflict of Interest

The authors declare that the research was conducted in the absence of any commercial or financial relationships that could be construed as a potential conflict of interest.
